# Biosignals reflect pair-dynamics in collaborative work: EDA and ECG study of pair-programming in a classroom environment

**DOI:** 10.1038/s41598-018-21518-3

**Published:** 2018-02-16

**Authors:** Lauri Ahonen, Benjamin Ultan Cowley, Arto Hellas, Kai Puolamäki

**Affiliations:** 1 Finnish Institute of Occupational Health, Helsinki, Finland; 2 Cognitive Science, Department of Digital Humanities, University of Helsinki, Helsinki, Finland; 3 Cognitive Brain Research Unit, Department of Psychology and Logopedics, University of Helsinki, Helsinki, Finland; 4 Department of Computer Science, University of Helsinki, Helsinki, Finland; 5 Department of Computer Science, Aalto University, Helsinki, Finland

## Abstract

Collaboration is a complex phenomenon, where intersubjective dynamics can greatly affect the productive outcome. Evaluation of collaboration is thus of great interest, and can potentially help achieve better outcomes and performance. However, quantitative measurement of collaboration is difficult, because much of the interaction occurs in the intersubjective space between collaborators. Manual observation and/or self-reports are subjective, laborious, and have a poor temporal resolution. The problem is compounded in natural settings where task-activity and response-compliance cannot be controlled. Physiological signals provide an objective mean to quantify intersubjective rapport (as synchrony), but require novel methods to support broad deployment outside the lab. We studied 28 student dyads during a self-directed classroom pair-programming exercise. Sympathetic and parasympathetic nervous system activation was measured during task performance using electrodermal activity and electrocardiography. Results suggest that (a) we can *isolate cognitive processes* (*mental workload*) *from confounding environmental effects*, and (b) *electrodermal signals show role*-*specific but correlated affective response profiles*. We demonstrate the potential for social physiological compliance to quantify pair-work in natural settings, with no experimental manipulation of participants required. Our objective approach has a high temporal resolution, is scalable, non-intrusive, and robust.

## Introduction

Cooperation and collaboration for mutual gain occurs throughout the natural world, from humans^1^, to animals^2^, to bacteria^3^. Collaboration tends to present a complex phenomenon: even in simple animal models where actions can be modelled individually, interpretation of motivations can remain unclear^4^. While in typically more-complex human studies one can use self-reports to query motivations, there remains another difficulty: results of laboratory studies (where self-report is feasible) cannot be assumed to apply in natural settings (where self-report could potentially interfere with the task).

Emerging ubiquitous measurement technologies, however, provide access to vast amounts of multimodal data to quantify teamwork and collaboration in natural settings such as workplace or classroom. Such measurements tend to be hard to interpret using methods traditionally employed for lab data. Specifically, we address the problem of how to derive insights from noisy psychophysiological measurements of collaboration in real-world tasks; that is, measurements taken without an experimental manipulation and control group.

Measurement and management of collaboration in programming is an area of growing study^5–8^ due to the commercial benefits of increased productivity. A study on pair-programming in education^9^ reported two factors that lead to successful pairings: a good match between partners’ level of programming skill, and a good match of skill level to the task’s demand-level. If these factors are badly matched, the collaboration can be poor in several ways: mismatch of skill can lead to inefficiency or frustration; excess demand can lead to exhaustion or intimidation; insufficient demand can lead to demotivation. Difference of programming experience within a team is relatively simple to track and manage. It is more difficult to deal with intersubjective synergistic factors, for example feedback dynamics and information sharing. Compounding the difficulty is that intersubjective dynamics occur during real-time task execution and cannot be interrupted to make observations such as self-reports or interviews. Understanding such collaboration requires reliable means to observe relevant aspects of the interaction, and clear understanding of how these aspects arise.

Specifically, pair-wise collaboration includes both the interaction of each worker with the shared task, and intersubject interactions. In programming, it is feasible to adapt existing work-monitoring tools to observe the interactions between worker and task, exactly as proposed by Bani-Salameh and colleagues^10^ with their social collaborative integrated development environment. At least for solo programming, such task-logging observations collected by software can be quite well interpreted on the group level using data-mining^11–15^. By contrast, the intersubjective interaction is not so easy to record as it occurs across a range of modalities from verbal to haptic^16^; and it remains quite difficult to interpret observations when experimental controls are relaxed. Yet, understanding this interaction is needed to understand the collaboration as a whole.

One promising approach relies on the well-established fact that certain aspects of the physiology of collocated interacting individuals will synchronize^17–19^, a phenomenon termed Social Physiological Compliance (SPC)^20^. However, merely establishing the presence of SPC does not *primae facie* inform us about intersubjective dynamics of collaboration — we must have methods that can show both that SPC occurs and also that psychophysiology is related to task in hand. Psychophysiology is complex to interpret, as it is sensitive to a variety of internal and external states, e.g., mental workload, fatigue, affective stress, as well as physical activity and for some signals, environmental factors such as ambient temperature^21^. Some issues can be solved by using multimodal instead of unimodal approaches^22–24^; however in general, extracting parameters that express the above-mentioned states in noisy physiological signals requires not only insight into human physiology but also strong data analysis know-how^25–27^. Until recently, such issues constrained the use of psychophysiology to lab studies; and although wearable devices are now common, interpreting physiology recording in natural settings without experimental control is highly challenging.

To study SPC we aim primarily to identify and isolate different types of stress^28^, which can be achieved reliably and robustly by measuring the various activations of different branches of autonomous nervous system (ANS)^29^. We aim on assessing collaboration based on synchronous arousal in dyads. Two signals which combine relative reliability of recording with good characterisation of ANS activity are electrocardiogram (ECG) and electrodermal activation (EDA). Thus, here we report a study of collaboration dynamics, in a protocol with completely natural task and setting, using SPC from ECG and EDA as an index.

Prior work on SPC has evolved from the lab to the real-world environments. Gottman set an early example, studying marital synchrony (perhaps the hardest work of all)^17^ using physiology including EDA; this line of study continues, e.g., Timmons and colleagues studied synchrony of cortisol^30^. For recent reviews, see Chanel and Mühl^31^, Delaherche and colleagues^32^, and especially Palumbo and others^33^. Studies in laboratory settings have used information in physiological signals to characterize collaboration of co-workers^34,35^, and predict collaboration outcome^36^. Selecting features of interest is of high importance must be examined carefully to reflect the desired character of the SPC^33,37^. In our own previous study, we presented a collaborative index^38^ using *tonic* heart rate metrics, such as heart rate variability (HRV), as a standalone index of SPC in pair-programming over the time-scale of a complete task (minutes to hours). However, the coarse temporal resolution of such an approach limits the potential to derive insights into collaboration dynamics. Although, very short interval HRV features have been introduced^39,40^, their reliability is yet unproven. An important open issue of cardiac SPC is whether given HRV activity arises from sympathetic or parasympathetic nervous system activation^41^.

While ECG provides valuable psychophysiological information at time scales longer than minutes, EDA provides much higher intrinsic time precision^42^: measurable EDA responses typically occur 1–3 s after a stimulus onset^43^. Such phasic sympathetic-arousal responses overlay a tonic level of conductivity, which varies slowly over minutes. Thus, methods to process EDA are also important – recent work on model-based EDA analysis^44^ has shown the challenge of correctly decomposing this signal into its tonic and phasic parts. While sympathovagal balance extracted from ECG signal might derive useful information, EDA reflects only sympathetic activation and may provide more robust insight into emotional valence^43^.

Fast, reactive, and robust physiological signals, such as spikes in EDA, have been studied for automatic unobtrusive affective state evaluation since early 2000s^45^. However functional models and systems of, e.g., emotion recognition are still under development^46,47^. The research on stress detection^48^ and affective state classification^49^ still relies on response induction and laboratory protocols for control. Using EDA in event related paradigms has traditions in music research^50,51^ and it has been used, e.g., in game research^52,53^.

Use of EDA to study collaborative work is growing. Multiple recent studies assessed collaboration in demanding tasks, though in laboratory settings^8,36,54^. For example, Xu and colleagues^8^ measured collaboration performance using EDA and personality traits in laboratory conditions while participants performed a classic demanding-task battery^55^. Mønster and colleagues^56^ studied team work in an experimental task setting, finding that synchrony of skin conductance was correlated with self-reported group tension and negative affect. In a recent study^57^, EDA was used to study collaborative learning, but specific features of the signal were not analysed; a crucial step to separate nervous system influences.

Naturalistic designs in ecologically valid environments are still rare. Slovak and colleagues^58^ explored the synchrony of EDA in everyday social situations, speculating that synchrony corresponds to emotional reactivity. Unfortunately, this interesting work again analysed raw EDA, with no decomposition. They used a technique from a study by Marci and colleagues^59^, who studied EDA synchrony between patient and therapist in a psychiatric setting.

The field of social neuroscience has also studied inter-subject synchrony (using the term ‘hyperscanning’)^60–62^. Brain imaging is arguably less scalable, reliable and unobtrusive than ANS measures (in our pilot testing, it interfered with students’ work). Still, such work is relevant in theory and methods. For example, a recent classroom study^27^, on electroencephalography (EEG) synchrony in class room environment, reported elevated physiological inter-personal synchrony in participants who made eye-contact prior to recordings.

Other methods to understand collaborative dynamics include exhaustive self-reports^63^; also used to provide context when for studies that extracted information from physiology^54^. This approach produces single-observation *post hoc* data points about the collaboration, which is problematic as it limits the information that can be gleaned from the continuous time series of, e.g., physiological measurement. Another approach has been observation (direct or indirect by, e.g., video recording) and annotation by a trained third party^64^. Experience sampling, originally developed in the study of Flow, can be used; as can post-hoc interviews^65^.

To bring collaborator-synchrony research to truly natural settings, for example in a school or an office, solutions must meet several requirements: unobtrusive, inexpensive, scalable, and descriptive of both the occurence and dynamics of synchrony. We argue that physiology measurement is highly scalable and cost-effective approach, once the methods are streamlined. Of these, ANS measurement is less obtrusive than CNS or head-mounted eye-tracking (remote sensing is a promising but still maturing area^25^). Hence, in this paper we study novel means to characterize collaboration using SPC measured via noisy signals from ANS, in uncontrolled natural settings.

To meet the last requirement and describe both occurence and dynamics of synchrony, we look at multiple timescales. Many existing approaches calculate features to represent SPC, such as correlation, coherence, cross-recurrence, or co-occurence, that are not sensitive to short-timescale transient phenomena^16,54,66^. Our approach is to establish the occurence of SPC using correlation of ECG features at 1+ minute timescales; then examine 1+ second timescale EDA activity of collaborators using both correlation and a confidence-band comparison method^67^ novel to this field. The precise timescale values are not of critical importance but merely match the order of magnitude of variation in the signal of interest, established in literature^68^.

We anticipate that results presented here should provide a clearer view on collaboration dynamics, to associate team scenarios with outcomes in realistic settings. We thus address the following research questions (RQs).

## Protocol and research questions

**Figure 1:**
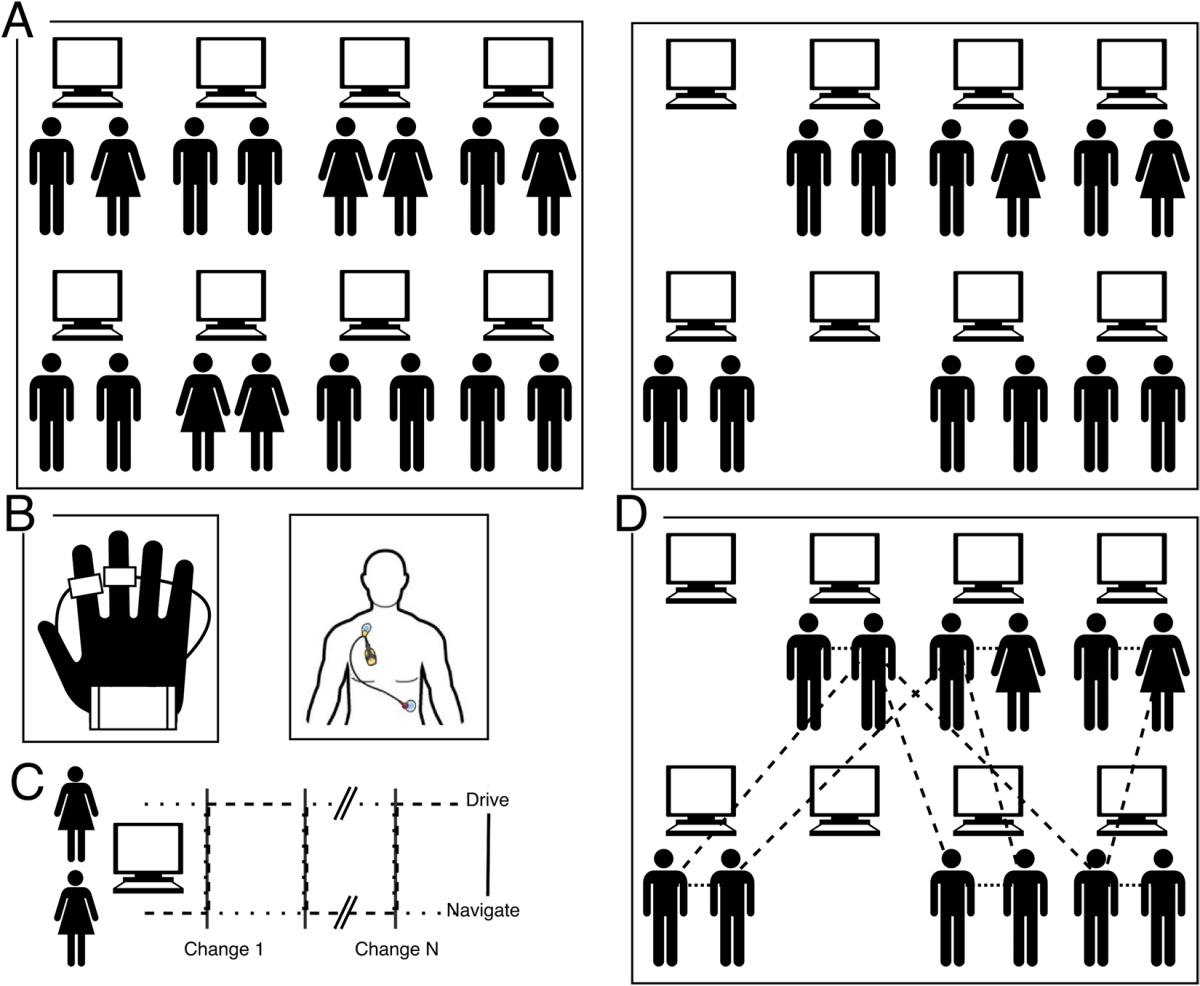
(**A**) The students were arranged in dyads seated at a shared computer, in a series of sessions with differing numbers of students per session. (**B**) Electrodermal activity (EDA) and electrocardiograph (ECG) were recorded as shown. (**C**) The task involved a series of assignments with periodic role switching. (**D**) For first research questions (RQ1&2), social physiological compliance (SPC) was compared between dyads (dotted lines) and the general correlation of the classroom (long-dashed lines), estimated via a bootstrapping procedure.

Our protocol is based on a conventional pair-programming task design used in education^69–71^; as such it represents a completely natural task activity for the participants. In pair programming, dyads of students work on a single work station while taking turns to play the role of navigator and driver, i.e., guiding through and actuating the ideas, respectively. The roles of driver and navigator change multiple times within a session, while multiple linked assignments are given and completed at the dyad’s own pace. The protocol, recording setup, and SPC analysis are illustrated schematically in Fig. 1, and described in detail in Methods.

In our previous study^38^ we showed that SPC, calculated from windowed HRV metrics related to cognitive effort, differs between co-working pairs and their collocated group. In contrast, the SPC calculated from windowed average heart rate (HR) and indicating physical activation, did not differ between co-working pairs and their collocated group. This result was based on a protocol with temporal parameters fixed across the group. Thus we redesigned the protocol to allow a more natural temporal structure, where the role-maintenance time remained the same, but participants now self-paced the role-switch duration (see Methods), introducing some drift into the cross-environmental synchrony. Temporal structure of knowledge work tasks is a logical target for increasing naturalism, as it may be assumed to be more worker-controlled than, e.g., content of tasks. We also introduce event-based analysis for specific events inside the sessions by automatic extraction of task-related events. We therefore ask.

## RQ1 Can we extract SPC from HRV variables in a natural protocol?

We expect physiological indicators of mental workload (i.e., HRV features) to synchronise more between collaborating students than the general level within the classroom; further we expect this to be in contrast to indicators of physical activation (HR). We expect visibility of the effect to be reduced given the decreased time synchrony between pairs, which also motivates study of higher time-resolution signals for SPC.

## RQ2 Can windowed HRV be substituted by fast biosignals for examining SPC in a natural protocol?

Signals with higher time resolution (under a minute) are potentially more sensitive to events with short time-scales, than signals which can only be calculated for 1-minute or longer scales (e.g., HRV). Thus, EDA should convey information on ANS activations in more detail than HRV features. Higher time-resolution should also enable better examination of collaboration dynamics, contingent on the presence of SPC in tonic EDA.

## RQ3 Can the physiological signals with high temporal resolution, found to reflect SPC, be associated with task related emotional valence and engagement?

The EDA signal has been linked to emotional responding^25^, and should thus be sensitive to events that elicit an emotional response during the programming task, such as success in running the assignment. Compared to the HRV features EDA responses are faster. Thus, the task dependence of the observed SPC could potentially be observed.

### Approach to RQs

We address the RQs with the following hypotheses and analyses (full details of procedures are given in Methods section). As in our previous study^38^, we define an index of HRV as the standard deviation of normal to normal heart beats (SDNN; normal heart beat is, e.g., not ectopic), calculated from the shortest-advised 60 second window (and the canonical 300 second window for comparison)^72,73^. Mean heart rate is calculated using the same windows. See Ahonen and colleagues^38^ for discussion about the window lengths.

We also attempt to reduce sources of noise by temporal filtering, i.e., by calculating indices from data in which the windows that overlapped with task-change have been ignored. However, due to frequent overlap between role-switching events and the longer analysis window filtering out these windows as well would have reduced the data by c. 80%. Thus only results from raw data and task-change filtered data are reported for the HRV feature analysis. Our null hypothesis is that the pairwise correlations of SDNN and mean heart rate between a collaborating students is indistinguishable from the correlation between randomly selected students in the same classroom. We obtain samples from the null distribution by a bootstrap procedure, described in detail in Procedures section. Thus we state hypothesis H1: *average correlation of SDNN in collaborating participants*’ *signals will be higher* (*exceed the upper bound of 95*% *confidence interval*) *than pair*-*wise SDNN correlations computed across the collocated sample*.

To investigate SPC of signals with response time within seconds, we used three features: skin conductance response (SCR) and skin conductance level (SCL) of the EDA, as well as instantaneous heart-rate (HR1). All three are sampled at one second resolution. HR1 is included solely for illustrating the effect of sample size. We can thus state hypothesis H2: *average correlation of SCR*, *SCL*, *and HR1 in collaborating participants*’ *signals will be higher* (*exceed the upper bound 95*% *confidence interval*) *than corresponding average of randomly selected pair*-*wise correlation across the collocated sample*. We use the identical bootstrap procedure for the fast signals as for SDNN and HR.

Finally, to explore natural collaboration dynamics in more (temporal) detail, we focused on interaction between SCR, student roles in dyads (drive, navigate), and meaningful task event outcomes (pass or fail a compiler test of the code). SCR was extracted in a short window around each event. Datasets were separated into four categories by the role in dyad and event outcome. The group-wise distributions of the SCR curves for each condition were estimated using bootstrap resampling.

Given this set-up, we state H3: *SCR locked to meaningful task event outcomes will show feedback*-*specific responses modulated by role of the participant*. Support for this hypothesis demonstrates the binding of the observed SPC to the task in hand.

In order to statistically test and visualise the grand average time series data, we employed novel methods, namely the minimum-width envelope (MWE) method. It has not been previously applied in this field, and requires a brief introduction (see Methods for details). MWE approach permits us to characterize the distribution of curves while controlling for the statistical properties of time series (e.g., non-independence of individual samples). MWE is a generalization of univariate confidence intervals to multivariate time series data^67,74^. MWEs thus retain the advantages of confidence intervals: they have an intuitive visual schematic because the true average of the distribution traverses inside the MWE; and the MWE gives a sound statistical interpretation, that is, the mean of the modelled distribution is fully inside the MWE with probability of 1 − *α*, where *α* is the desired level of control of Type I error. We have used *α* = 0.05 throughout this paper.

MWE is defined by lower and upper bounds of the signal mean at each time point, respectively. To use MWE to test whether samples from two conditions are drawn from separate distributions, the most convenient and powerful approach is to obtain difference curves by subtraction and calculate their MWE. If *at any point* the consequent zero is outside the MWE, it shows that the curves as a whole are statistically significantly different.

Therefore, to illustrate the interaction of SCR with roles and event outcomes, we plot the means for two conditions against each other, and alongside we plot the MWEs of difference curves for each condition contrast.

An additional approach to explore high time-resolution collaboration dynamics was also studied. The difference of each signal and itself lagged by ten seconds was computed for each participant and grand averages were obtained for each role × outcome condition. The variations in the change signals were obtained using bootstrapping for participant-wise averages. The confidence band was again estimated using MWE method. This would further expose event related changes in participant’s psychophysiology.

## Results

### RQ1: tonic HRV-based SPC

Table 1, rows 1–4, show the dyadic and classroom correlation of SDNN and mean HR in 60 and 300 second windows. Rows 5–8 show the same time windows for filtered data where tasks changes have been removed. For the SDNN index of HRV in short time windows, the average SPC of collaborating participants is higher than the upper bound of the 95% confidence interval for the collocated sample’s SPC distribution. This result is only marginally significant when SDNN is calculated from all data, but is significant *p* < 0.05 when task changes are controlled. *p*-values are adjusted for family-wise error rate over all signals in the Table 1.

**Table 1:** Mean correlations of collaborating pairs and their confidence intervals under null hypothesis that the correlation is independent of the pair assignment within the class: heart rate and standard deviation of successive differences (SDNN).

HRV feature	window (s)	avg. cor. in dyads	95% ci	adj. p-value
Mean HR	60	0.27	[0.12, 0.29]	0.32
SDNN	60	0.15	[0.04, 0.14]	0.07^•^
Mean HR	300	0.31	[0.12, 0.40]	0.53
SDNN	300	0.15	[0.03, 0.22]	0.53
Results with data where task-switch times are removed				
Mean HR	60	0.34	[0.13, 0.34]	0.14
SDNN	60	0.17	[0.05, 0.15]	0.04*
Mean HR	300	0.42	[0.15, 0.45]	0.32
SDNN	300	0.23	[0.05, 0.28]	0.39

Statistical significance is denoted by *p < 0.05 and ^•^*p* < 0.1. *p*-values were corrected against family-wise error rate.

Table 1 also shows that windowed mean heart rate (HR) is not significantly different between collaborators and overall correlations in each classroom. The average correlation of the HR is higher than for the SDNN, as in the previous study; this effect is partly caused by the decrease in HR from the beginning of the session^38^.

### RQ2: tonic SPC of signals with time resolution of one second

Skin conductance response and level (SCR and SCL, respectively) and instantaneous heart rate (HR1) results are presented in Table 2. In a similar manner to the separation of cognitive (SDNN) and physical (mean HR) activations in the previous paradigm^38^, here the faster, mental-state reactive feature of EDA (SCR) separates pairs from group, while the slower, baseline-physical-state feature of EDA (SCL) does not. The average correlation for collaborating pairs in full series of raw HR1 is increased from randomly drawn sample as well.

**Table 2:** Mean correlations of collaborating pairs and their confidence intervals under null hypothesis that the correlation is independent of the pair assignment within the class for the more time precise signals: skin conductance response (SCR), skin conductance level (SCL), and instantaneous heart rate (HR1).

Signal	avg cor in dyads	95% ci	adj. p-value
SCR	0.12	[0.04, 0.09]	<0.01*
SCL	0.38	[0.26, 0.44]	1.00
HR1	0.13	[0.04, 0.10]	<0.01*

The collected signals were downsampled to 1 second aggregates to match the event-logger time precision. The *p*-values are corrected against other fast signals.

### RQ3: SCR around interesting events

**Figure 2:**
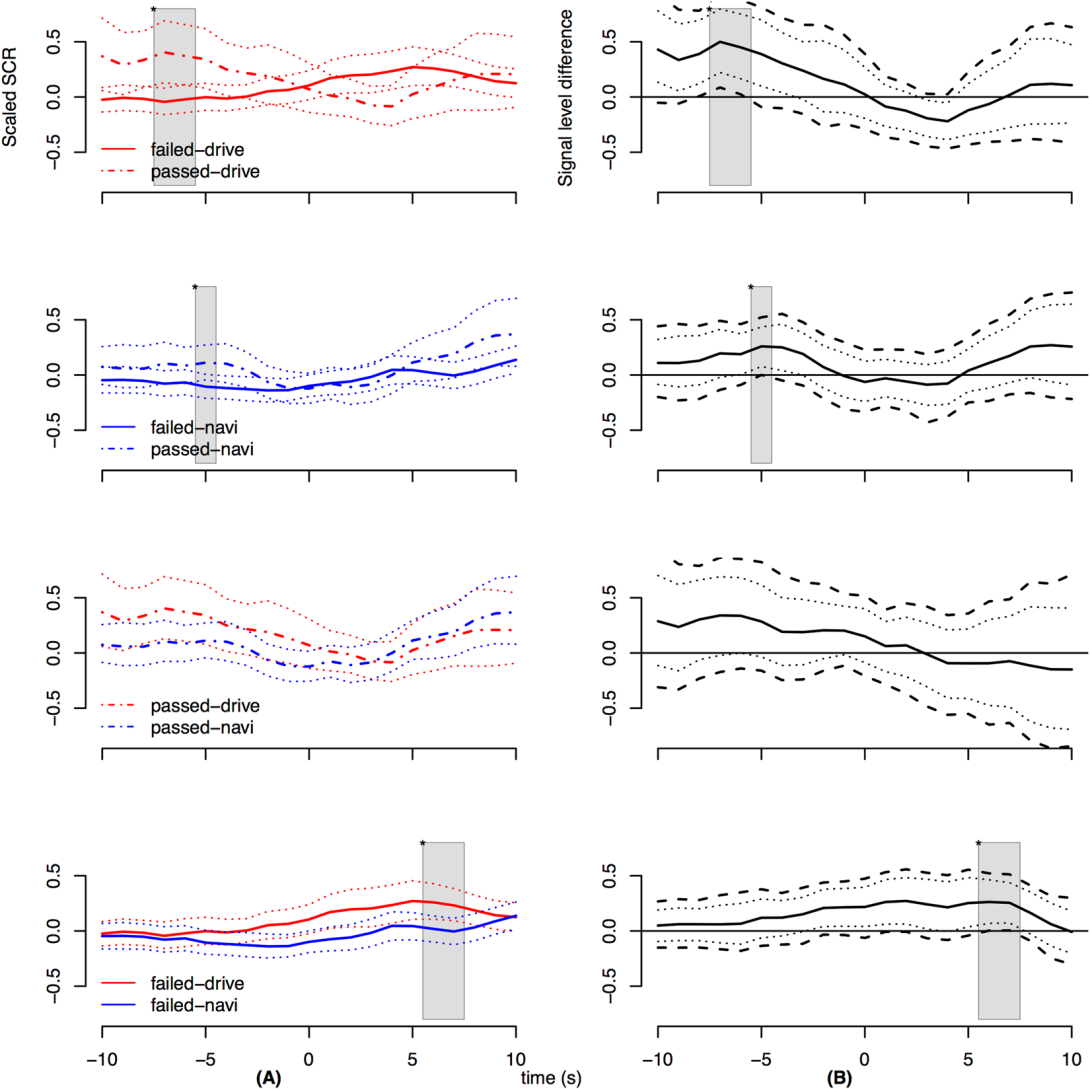
(**A**) Left: The grand average of normalized skin conductance response (SCR) in driving and navigating roles with different outcomes in events. The two top rows of the left column illustrate the differences in passing and failing (line type difference) around running and testing events (time 0). The lower two rows of the left column illustrate effect of roles (colour difference) around the events (time 0). Dotted lines show naive *p* < 0.05 bootstrapped confidence intervals for each time point. (**B**) Right: The right column shows the grand averages of intra-participant differences in scaled SCR signals, corresponding to the respective plot in the left column. The participant-wise averages were sampled to find confidence intervals for the differences. Dashed lines show confidence bands derived using MWE^67^ corrected boundaries for *p* < 0.05 significance. Dotted lines show the naive 95% bootstrapped confidence intervals for each time point. The gray background marks time points where the signal level difference deviates from zero significantly.

Figure 2 illustrates the SCR signal levels (left column) and signal differences (right column) for the contrast of each combination of role (drive/navi) and outcome (failed/passed) of the events in the analysis, i.e., the occurences of compiling or running the code. Significant difference of the curves is seen by non-overlap *at any point* of MWE bands in right column (by band-deviation from zero), as illustrated by gray areas in Fig. 2. For comparison, the naive 95% bands of the estimated bootstrap distributions are also plotted (dotted line in both columns). Note that the confidence bands are wider for pass events due to smaller number of samples in participant averages.

By visual examination, the means for different-outcome same-role have shapes which suggest they are out of phase, i.e., means rise and fall at different times (Fig. 2, left column, rows 1 and 2). On the other hand, the means for same-outcome different-role tend to rise and fall at similar times (Fig. 2, left column, rows 3 and 4).

Considering significance tests, Fig. 2 top row reveals a significant difference between failed and passed events in driving-role grand averages due to −$8\cdots -6$ seconds difference before the events. Here the outcome curves have strongest out-of-phase morphology. In the second row, for navigating-role averages, the signal level difference is still significant as found from 5 seconds before the events.

The bottom row of Fig. 2 reveals a significant difference in signal levels between roles in failed events due to difference at $6\cdots 7$ seconds after the events. Overall the morphology shows that drivers react sooner and with greater peak amplitude than navigators. Similar effects cannot be found in difference curve for passed events (third row), where morphology is quite similar between roles.

Overall Fig. 2 suggests that (a) signal levels are much higher before the events when the test/run is going to pass compared to when it will fail; and (b) for failed test/runs, signal levels increase more when participant is in driving role compared to navigating role.

#### Exploring time lagged difference in signals

**Figure 3:**
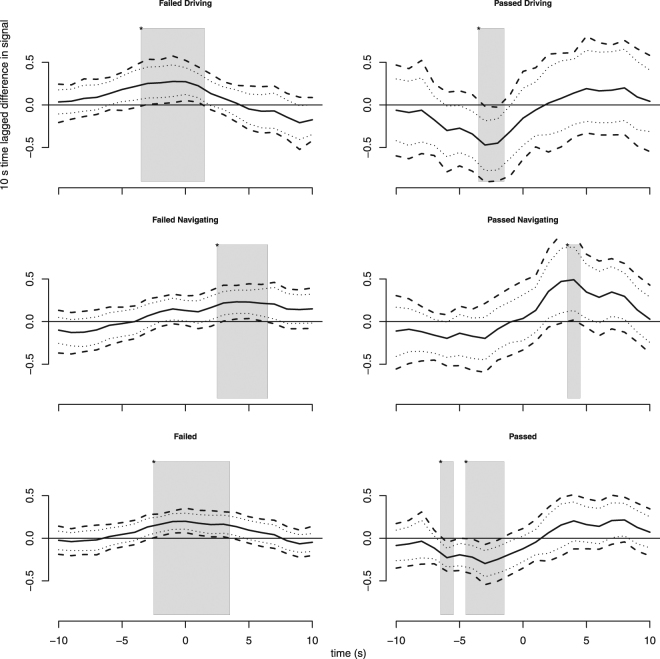
The grand averages of SCR 10 second time-lagged difference in time windows of −10 to 10 seconds. The figure illustrates the change in signal levels in different conditions. Times of significant signal change have grey background. Dashed lines show confidence bands derived using MWE^67^. The MWE’s contain 95% of the bootstrapped difference curves. Dotted lines show naive univariate 95% bootstrapped confidence intervals for each time point.

Figure 3 illustrates the SCR signals time lagged difference, plotting the mean, MWE, and naive 95% confidence intervals for the distribution mass of the signal subtracted from itself with a lag of 10 seconds.

Comparison of the plots in top row and middle row shows that in driving role the signal change manifests stronger and earlier when compared to the navigating role.

The bottom row reveals that when the role difference is not considered the fail event causes signal levels to rise from 2 seconds before to 5 seconds after the event and decrease from 5 seconds before until time 0 for passed events. These findings support the interpretation of the plain SCR signal averages in Fig. 2.

## Discussion

In the present study we examined the utility of ECG and EDA signals for assessing collaborative behaviours in a natural setting. We found that features of HRV and SCR provide a reliable basis to calculate an SPC index of collaboration. Furthermore we showed that SCR contains information on collaboration dynamics and different team functions. Finally, SCR also provided insight into the valence of meaningful task events.

Addressing RQ1, we provided evidence that the SPC index based on correlation of SDNN was significantly higher between pairs than the chance level in 60 second windows. This reproduced the earlier results^38^ though with smaller effect size, in an updated setting with shorter natural collaborating periods (more available tasks) and self-paced role switching. We did not replicate the earlier result for the 300 second time window. This was probably due to the mentioned changes in activity structure, because correlation in collaborators’ signals increased when task-change time instances were left out of the analysis. This was further supported by also removing the windows with role-switches, but then data was reduced to a fraction of the full dataset with longer time windows. Thus it was considered not reliable result and is not reported above.

For RQ2, the significant correlation for collaborating dyads in instantaneous HR1 and SCR shows that *SPC of co*-*workers in natural settings can also be derived from fast signals*. The signals chosen were motivated by the setting. For example, eye tracking has been used to predict collaborative performance in a laboratory study^54^; but this signal is difficult to bring to a natural setting^25^. There are therefore only a limited number of modalities that are suitable for investigating short-term phenomena in cognitive aspects of collaboration, among which HR1 and EDA are easily measurable, moderately resistant to artefacts, and well-documented^75,76^.

The seeming contradiction of HR1 expressing correlation increase for pair-work while windowed mean heart rate does not is explained by the nature of HR1 carrying information of both, mean and variance, i.e., heart rate and SDNN, of the ECG signal^41,42^. However, HR1 would be unsuitable for examining, e.g., sympathovagal balance^41^, as this index is extremely sensitive to movements. Also, while good for recording purposes, HR1 reflects innervation of both sympathetic and parasympathetic branches of the ANS^41^. Windowed calculation of HR and HRV helps to separate sources of activation^38^.

When EDA is separated into SCR and SCL, we obtain a similar separation of influences as given by the HRV/HR division. Thus the RQ2 results signify a similar pattern of SPC among collaborators as previously reported by^38^, where SDNN was associated with cognitive requirements of collaboration. SCR has also been reported to conduct information on cognitive load^77^, collaboration^8^, and emotions^78^.

The interpretability of EDA features implies that they can be used alongside HRV features to study collaboration at group level. Moreover the effect size obtained was large whereas the effect size of HRV results in this study remained small. Combined with RQ1 results, this suggests our first main finding: SPC can still be observed from readily-measured biosignals recorded during collaborating periods, even in a paradigm with shorter, natural pace.

The demonstration of fast-signal SPC in the results above is also a necessary condition to support the further investigation of RQ3. RQ3 results for difference of pass/fail events revealed that valence can be found in signals. Further, in both roles the results show that main difference lies in the *anticipation* of the event outcome.

Role differences in signals seem to indicate utility of SCR in examining how the effects of stressors vary inside a pair, namely, the failure outcomes are significantly more arousing for drivers than navigators. This might reflect engagement, or the liability of being in the leading role.

The event-based analysis of SCR focused on *run* and *test* events, which respectively indicate that the code compiles without errors, and whether or not the code passes instructor-assigned tests. These are *a priori* meaningful events: no matter the context or outcome, pair-programmers (in either role) should be engaged by these events (because they are fundamental for the completion of the assignments). Furthermore, it should be noted that the difference between *run* and *test* events is not explored, precisely because the event classes are not of primary interest. By combining the event types more statistical power was achieved. It appears that as long as they are *a priori* meaningful, any event can elicit pair dynamics in physiology. On this basis the approach should generalise well to other domains with meaningful events; as noted earlier, music and game play are two such domains^50–53^.

Examining the temporal evolution of the signals from different conditions, Fig. 3, helps to illustrate the varying reactions in different roles and outcomes. For fail events, drivers show a significant increase in SCR up to event time (giving difference curve above zero), whereas navigators are lagged by several seconds. This may indicate that drivers are more in tune with their code than navigators, expecting the failure. This finding is also supported by earlier literature on collaborative psychophysiology^79,80^. In contrast, for pass events the driver difference curve confidence band drops below zero around −2 seconds, before rebound. This indicates that drivers are aroused before the pass event, possibly due to uncertainty about the goodness of their code, and then relax while the event passes with positive result. The navigators on the other hand, do not show a similar dip up to event time, instead they show a strong rise after the event, indicating that they perhaps did not maintain an ongoing mental model of code goodness but are still interested (and thus aroused) by (rarer) positive outcomes. These differences are probably also affecting the collaboration indices measured from the signals.

The difference curves for fail and pass events, combining both roles (Fig. 3 bottom row) summarize the distinction in outcomes: failed events induce arousal around the event itself, while pass events show that programmers had decreasing arousal before the event. This suggests the influence of reactive frustration and anticipatory relief, respectively. In sum, RQ3 results suggest that collaboration success/failure outcomes are possible to extract from real teamwork environments.

In general, our combination of methods to study event-locked SCR provides evidence that moves the state of the art one step closer to collaboration measurement in the wild. EDA is valuable here because it is a fast-responding signal but still stable and interpretable, unambiguously reflecting sympathetic activation. The choice of events is easily justified without reference to a complex model of neural processes. And the MWE method provides means to examine the dynamics in clear unambiguous detail, while controlling for the autocorrelation structure of time series data. Note that the MWE method can also be applied without relying on the bootstrap, based on permutation testing, as discussed in Methods and demonstrated in Supplementary material. This circumvents concerns about the assumptions required to use the bootstrap. The MWE thus allows us to better see the full picture, by examining more of the distribution information than central statistics. This has been described as highly valuable, for example when studying reaction times^81^. That the results we see are quite as expected (i.e., driver response comes first), lends credence to the validity of our approach.

Slovak and colleagues^58^ provided a *post*-*dictive* hypothesis that emotional reactivity is the basis of EDA synchrony. If this is true, it implies that in our study SCR synchrony among pairs was higher than chance because of emotional reactions to stimuli. This seems to be shown by the MWE results, given that both roles responded to events, with different delays. This could lend support to the Slovak hypothesis, although of course we did not set out to test the hypothesis and thus do not make an evidential claim.

The MWE results in Fig. 3 show a difference in lag between driver and navigator emotional responding to pass and fail events. This lag suggests that drivers have greater anticipation of the events, which ties in with the concept that programmers develop an extensive mental model of their work^82^. This mental model means that interruptions can be costly, but our results show that in a pair programming scenario, the navigator is not as likely to suffer such an interruption cost. Thus we suggest pair programming as a solution to the problem posed in Zuger and Fritz^82^: let the navigator act as an interruption buffer.

**Figure 4:**
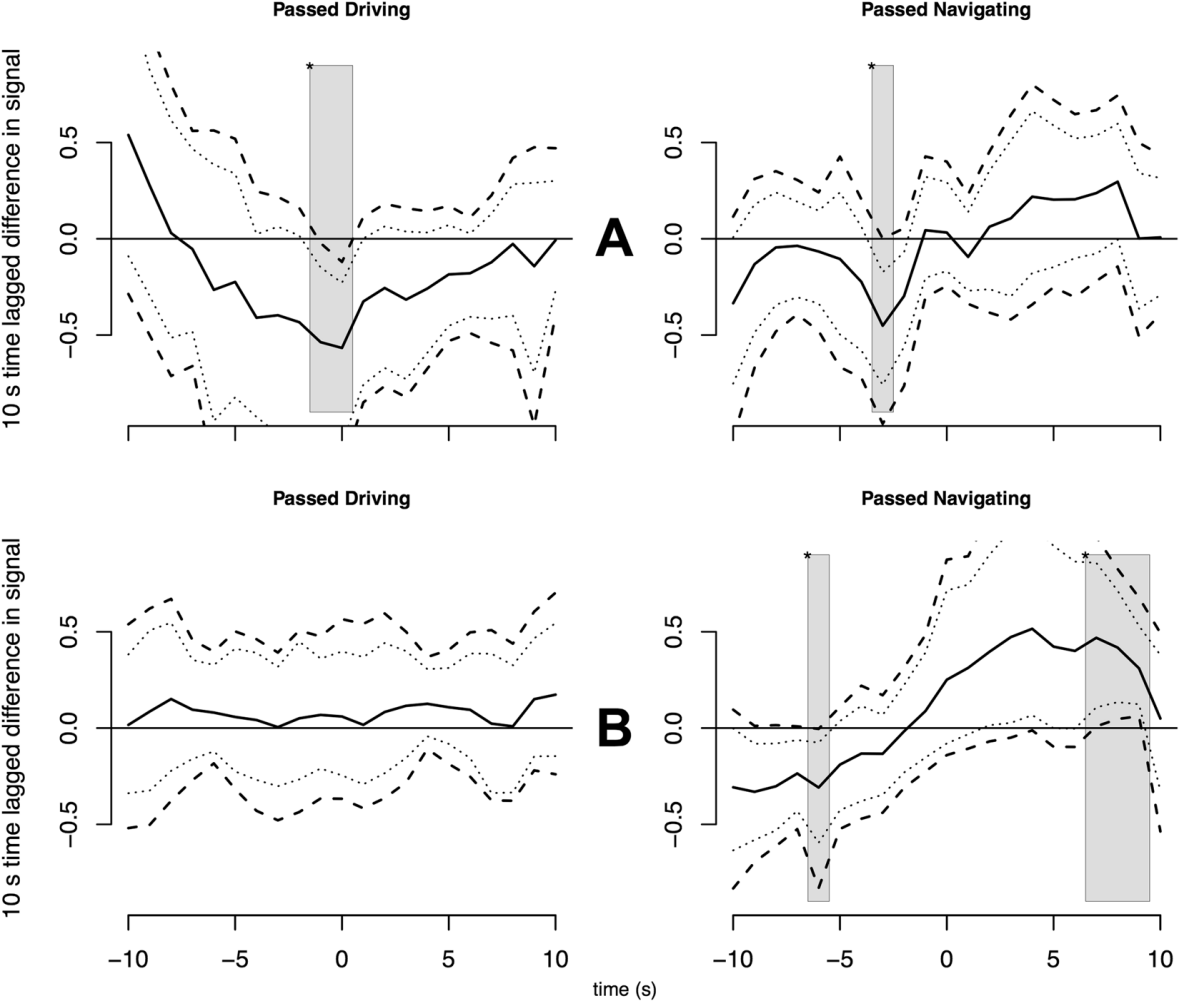
Using same method as for Fig. 3, SCR time lagged difference in curves for Passed-outcome × role conditions, using separate data from room 1 (top row) and room 4 (bottom row). The most interesting differences between rooms are in the conditions shown.

Regarding the utility or applicability of our method, one obvious use-case is to compare the SCR MWE of naturally-occurring groups. To illustrate this idea, we next perform such a comparison between the individual rooms of pair-programming students in our sample. We examine two rooms of the study to check whether the same SCR/MWE-based examination shows useful information for individual groups. These groups are not large, with room-wise *N* = [8, 16], and so the statistical power is insufficient to include room-wise analysis as a research question. Nevertheless the numbers suffice for an exploratory question. To this end, we make a visual inspection similar to Fig. 3 above, for rooms 1 (*N* = 14) and 4 (*N* = 16) in successful events only (see Fig. 4).

As we see, several conditions show characteristic variation from each other. For example, the ‘Passed Driving’ condition in room 1 (panel A) roughly follows the morphology of the main data (though with a noticeably steeper descending onset and a later point of significance), but for room 4 (panel B) the SCR in this condition is almost flat. On the other hand, for the ‘Passed Navigating’ condition, both rooms follow the morphology of the main data: but room 4 does so with a steeper positive slope from pre- to post-event time, with MWE lying outside zero both before −5 and after 5 seconds.

For a user interested in applied questions, such as a course instructor, this could be taken as motivation to examine the participant groups of the two rooms separately. Looking at the self-reports obtained in the experiment, one difference in the rooms occurs in response to the post-test Nasa TLX item on level of frustration experienced (see Methods, and Table 3): it is (non-significantly) higher for room 1 and than room 4. Thus the drivers in room 1 may have been more emotionally invested in their run/test events, and consequently experienced stronger relief after a Pass, than in room 4. This might speak to the respective skill levels, or to a more subtle effect due to the relationship between collaborators. Further work is needed to make such questions answerable.

**Table 3:** Frequency statistics for self-reports: number of participants responding at each point of each scale.

	0	1	2	3	4	5	6	7	8	9	10
Mental demand	0	1	3	8	11	8	12	4	5	4	0
Temporal demand	0	0	4	4	4	25	5	5	5	3	1
Performance	1	0	0	2	2	3	8	11	11	8	10
Effort	0	0	2	7	3	3	15	16	5	5	0
Frustration	2	8	9	8	7	8	3	6	3	2	0
KSS	—	0	14	12	16	3	8	2	1	0	—
Following coworker	0	0	0	1	0	1	3	9	16	14	12
Coworking history	40	4	1	1	0	2	0	2	1	1	4

Self-report items include the Finnish translation of NASA-TLX questionnaire (excluding the ‘Physical demand’ item), rows 1–5; and the Karolinska Sleepiness Scale (KSS), row 6. Participants also reported their common collaboration history (Coworking history) and the collaboration effort during the session (Following coworker), rows 7–8. All the TLX responses were given in 11-point scale from 0 being very low to 10 being very high except “How well did you perform?” (Performance) ranged from 0 failure to 10 perfect. KSS was reported in normal 9-point scale from 1 being extremely alert to 9 extremely sleepy. The coworking history scale ranged from 0 being no collaboration to 10 very much collaboration.

Given the ability of our method to highlight sub-group valence responses (Fig. 4), it is interesting to note the inverse relation between EDA synchrony and team-wise negative affect found by Mønster and colleagues^56^. Our additional analysis with sub-group averages also suggest that questionnaire results were inversely related to EDA responses.

Given these results, knowledge work is a potential example where the communication and collaboration quality between personnel is of measurable value, where projects can often involve large amounts of computer-based, complex specialized tasks requiring input from multiple individuals. For instance, production and maintenance of software has high cost and societal value. The proportional cost for maintaining software now represents more than 90% of its total cost^83^. Approximately half of this cost involves understanding the code to be maintained and can be partly attributed to collaborative processes such as code review.

Our study has arguably two key limitations. Firstly, some subjects were involved in more than one session, causing a non-systematic form of repeated measures. Specifically, our paradigm was constrained to prevent formation of any dyads with the same members across sessions. However we did not constrain individual subjects from participating in more than one session because they would not be so constrained in their normal coursework activity. Thus, although the pair composition is different for all measures (implying that correlation features were not affected), there is an issue when single subject averages are analysed. Such repeated measures are not modelled in the event-based analysis for the following reasons. One, they represent a small proportion of the group averages which we are interested in. Two, there are other minor random effects between recording sessions which are lacking in the models as well. These include: varying physical properties of the electrode setup, changing environmental properties in the classroom, completely different tasks per session. Such effects can be viewed as noise which is expressed in our approach by the signal variation shown by the confidence bands. One could test the effect size of such repeated measures, but because we initially believed that our recording setup should be natural and not control repetition, unfortunately we did not track the repeating subjects. Thus this limitation could be handled in future paradigms.

The second limitation lies in temporal resolution of the features extracted for HRV-parameter correlation analysis. Windowing is necessary in order to capture relevant properties of underlying neural activations. As explained earlier, the shortest possible time window for calculating HRV features is about 60 seconds^72^. Also, we used overlapping time windows instead of sliding window analysis to prevent oversampling. For correlation analysis the samples are assumed to be independent inside the series. When the overlapping is increased also the auto-correlation increases and averaging can no longer effectively reduce the error variance^84^. Thus we used sparse 30% overlap, which results in reduced temporal resolution of the extracted HRV features compared to sliding windows. Notice that the MWE method controls for the effect of autocorrelation, whether it is due to natural autocorrelation in the signal or the overlapping windows.”

We reserve for future work all questions around predicting collaboration outcome using SPC. To investigate group level performance changes together with physiological parameters, one would need either: predetermined grouping for the assignments; systemic manipulation of difficulty as paradigm parameter; or longitudinal design to track differences between teams.

Together with previous results the study suggests that the measures of subjective performance are too noisy to classify in individual level despite the group level SPC measures showing robust indices of collaboration. Group analyses could however provide important information, for, e.g., extracting information about team performance on average. These population level (nomothetic) versus individual level (idiographic) analyses and methods are further discussed in review by Palumbo and others^33^.

Furthermore combining EDA and HRV could produce a more complete picture of ANS responses than either alone^85^. Earlier, specific activation of HRV has been explored by co-measuring endosomatic skin response measures (skin potential response)^29^ using EDA. These measures, known to be activated only by sympathetic branch of ANS, allow the responsiveness of HRV to be assessed while mainly reflecting changes in one part of ANS. By building a natural paradigm with appropriate time structure, this could provide more robust measures while still retaining affective valence markers.

Moreover, as SCR shows promise as a tonic feature of SPC, a predictive model could be built if a real classification task is introduced with training and testing datasets. The SCR phasic reactivity also opens opportunities to investigate initiative and lead-lag relationships, a topic of great interest in the field. For example, lead-lag relationships in SPC are important in inter-dyad dynamics, e.g., the cycles of eye-to-eye contact^54^ and aversion have been reported to relate to conversation turn-taking^17^. More sophisticated methods that would also model the directions of the causality within collaborating units could be introduced. This could potentially have abundant applications in education and the workplace.

In conclusion, the suitability, temporal resolution, scalability, and cost effectiveness of methods and designs used to study collaboration could be greatly improved in the long run, as called for by Palumbo and others^33^. Our results move toward this goal, illustrating the value of SCR as an index of SPC. The observed results are possible only due to the higher temporal resolution and interpretative clarity compared to other signals researched, and (for event-related results) only given our novel application of statistical methods that preserve temporal structure (i.e., MWE). As is, our findings should be of great interest to organisational and human resources research, they can support extremely valuable tools for management and monitoring of organisation level well-being. Additionally the valence-arousal associations found in SCR, reflecting only the sympathetic branch of ANS activation, work as group level measures and can thus be employed in future paradigms with more applied designs.

## Methods

### Participants

The study has been approved by the ethical review board in Humanities and Social and Behavioural Sciences of University of Helsinki, Finland. The guidelines of the Declaration of Helsinki for human experiments were obeyed.

The sample was drawn from freshmen programming students, recruited via class mailing lists, and remunerated with cinema tickets. In total 30 student pairs were recorded. Two of the participants needed to be excluded from the analysis due to the quality of EDA signal, each invalidating a single pair. The final data consisted of 56 datasets (28 pairs). The pair coding sessions were conducted in the same class room environment on five separate occasions, starting between noon and 14:00, as part of normal curriculum of the programming course. The participants in the final dataset consisted of 18 females, 20 males; 3 left-handed; age ranging from 18 to 41 years (mean 23). The sample is less than the number of datasets because some students attended more than one session. Because pairs were constrained to be unique (repairing was not allowed), and because each session had different assignments, we did not treat this as a case of repeated measures.

### Protocol

The protocol was similar to our previous study^38^, tackling program course assignments in a conventional pair coding schema^69–71^. Changes to the previous were made to the task structure in order to (i) decrease temporal synchrony thereby increasing naturalism, and (ii) increase the frequency of self-ratings for task performance (see below). Unfortunately, due to system errors, self-reported performance was recorded by only 61% of the participants.

Experimenter-guided seating was used to prevent pairs from self-selecting the collaborator (according to, e.g., their social preferences), and to improve intra-pair skill-matching (skill was estimated from self-report and course instructor’s feedback). Each pair worked on one workstation with a single keyboard and mouse.

Participants were first briefed, after which they provided written informed consent and the sensors were attached. Physiological measurements were started while participants launched their integrated development environment (IDE) (NetBeans for Java https://netbeans.org/). The first activity of the session was a seven-minute baseline video with class room lights dimmed, which allows the physiology to ‘settle’ from the initial exited state due to sensor attachments and abnormal class initiation. The subsequent programming exercises were standard assignments for the course, previously unseen by participants. In all sessions, total experiment time was about 90 minutes.

Every seven minutes the IDE prompted for role change. The roles swapped between ‘driving’, i.e., active programming using keyboard and mouse, and ‘navigating’, i.e., guiding and commenting. The time consumed to role change itself was at participants discretion, i.e., the new driver clicked when ready to start the next seven-minute period.

Pairs were assigned four to six programming exercises, consisting of several subtasks leading to one concluding main task, to be completed at participants’ own rate. The subtask exercises were counter-balanced so that if half of pairs in a session were presented with, e.g., subtasks 1–2–3, then the complement was given subtasks 2–1–3. Pairs needed to solve all the preceding subtasks before the final concluding task. In case of problems the participants got help from the assisting teacher in the class.

Before the session, participants completed the Karolinska Sleepiness Scale (KSS), and indicated (a) their sleeping time during the previous night (average 7 h:19 m, sd 1 h:44 m), and (b) hours spent awake before the experiment (average 5 h:58 m, sd 1 h:33 m). The results suggest a normal level of alertness and circadian physiology in the sample.

At the end of the session participants filled the NASA Task Load Index (TLX)^86^ form. Participants were also asked to report the history of collaboration with their assigned co-student; and to rate collaboration quality throughout the session. The distributions of these self-reports are shown in Table 3.

Two physiological signals were measured: electrocardiography (ECG) and electrodermal activity (EDA), as described in detail below. The participants were instructed to ignore the experimental apparatus insofar as possible.

### Analysis overview

To address RQ1 we recorded and analysed HRV features in similar manner to our previous study^38^. To address RQ2 we recorded EDA, extracted its main components, and performed similar analysis as for RQ1, to compare to the ECG results. Finally, to address RQ3 we extracted participant-wise SCR around specific programming events called “run” and “test” events, i.e., the moments when the pair was running or testing the code for evaluation purposes. We compared these events between conditions for different pair-programming roles, and failed or successful outcomes of the event.

### ECG

For measuring ECG signals we used eMotion Faros 180° devices (Mega Elektronikka Oy, Kuopio, Finland). The devices recorded 2-channel ECG signal with 500 Hz sampling rate. The devices were mounted near dominant shoulder under the collar bone. The ECG electrode placements were on right coracoid process and on the lower left rib-cage. The Faros device clocks were pre-synchronized with the pool.ntp.org virtual cluster of time-servers. From ECG signal the R-peaks were automatically detected with the Colibri package^87^ for the R platform for statistical computing^88^ and used to form the inter-beat interval (IBI) series, the basis of the HRV analyses. Preprocessing and extraction of the features (HR, SDNN) was also performed with Colibri. The features were computed according to their standard definitions^73^.

#### ECG analysis

HRV features were calculated from IBI series to address RQ1. The data was windowed to 60 second (shortest reliable HRV window) and 300 second (canonical 5 minutes window used widely in clinical applications)^72,73^. The successive windows overlapped by one-third (20 and 100 seconds respectively) providing more statistical power for the analysis compared to adjacent windowing.

For a given window length, we obtained two feature vectors for every participant, denoted ${{\bf{x}}}_{{\bf{i}}}^{{\bf{H}}{\bf{R}}}$, and ${{\bf{x}}}_{{\bf{i}}}^{{\bf{S}}{\bf{D}}{\bf{N}}{\bf{N}}}$, where *i* ∈ {1, 2, …, 56} is a participant identifier. Elements in the vectors were then upsampled to produce HRV values for every second. For every feature, *x*_*i*_(*t*) denotes the value of the feature in the second *t* from the start of the baseline video. This resulted in four features (HR60, SDNN60, HR300, and SDNN300) for each participant.

The feature vectors were compared using average Pearson’s product-moment correlation coefficient in permuted sets of vectors to the correlations in real collaborating participants’ signals. More specifically, the sets of two signals, denoted by identifiers (*i*, *j*), where *i* ranges from 1 to 28, and *j* = *i* + 28 were examined for correlation. The collaborating participants are denoted as (1, 29), (2, 30), … (28, 56). The values of $cor({x}_{i}^{F},{x}_{j}^{F})$ for every pair, where *F* is either HR or SDNN, are arranged to result ${\tilde{x}}_{true}$, that is the arithmetic mean of these pairwise Pearsons’ correlation coefficient. The ${\tilde{x}}_{true}$ is used as an estimate of SPC for collaborating members of pairs. The values of HR and SDNN correlations within collaborating pairs for window lengths of 60 and 300 seconds are shown in Table 1 column “avg. cor. in dyads”.

For isolating the SPC from general environmental effects we used permutation tests. Our null hypothesis was that the correlations within pairs do not differ from correlations in randomly chosen signals in each class room. This was tested with permutation, using shuffled pairs of signals within each class room. More formally, the permutation *r* is drawn *uniformly at random* to form a shuffled signal pair (*r*_*i*_, *r*_*j*_). The random pairing was formed with restriction that the signals were recorded in same session, i.e., in same class room. The sets of 28 shuffled values were drawn from the correlation matrix to match the number of observed compliances. The arithmetic mean of these sets, denoted *μ*_*r*_, was used as a sample from the null hypothesis.

The *μ*_*r*_ was computed 10000 times to estimate distribution of average correlations across participants in similar conditions. Any correlation that we observe for the the pairs sampled from null hypothesis should be caused by confounders in the environment instead of SPC. The obtained distribution sets confidence intervals for the *μ*_*r*_ and allows us to compute our one-tailed p-values for the true correlation averages (${\tilde{x}}_{true}$)^89^. Finally the p-values were corrected with Holm-Bonferroni method, including all computed p-values in the HRV analysis.

For comparing the results to our previous study^38^ we repeated the process described above but removed the samples with forced behaviour from the computations. Forced behaviour is found from the parts of the session when the pairs were changing roles (between driver and navigator) or when the subtask was ended and self-rating window appeared (and was filled). The analysis was conducted separately for only removing subtask changes. Additional removal for also task-switches reduced the amount of data with longer window below 20% of the original. Hence, these results were not reported.

Additionally instantaneous HR (HR1) was estimated from ECG data by the inverse of the average IBI length for each second (see section below for usage).

### EDA

For recording EDA we used Shimmer 3+ GSR devices (Realtime Technologies Ltd, Dublin, Ireland). The galvanic skin response (GSR) was recorded at 51.2 Hz sampling rate. The device was mounted on wrist of the non-dominant hand (opposite to mouse hand). The EDA (BlueSensor wet gel, Ag/AgCl) electrodes were attached with adhesive tape and gel on medial phalanxes of index and middle fingers in the non-dominant hand^42,90,91^. The Shimmers were synchronized before measurement sessions to the time of pool.ntp.org time-servers.

#### EDA analysis

The collected EDA data was subjected to analysis by first detecting and correcting motion induced artifacts through interpolation and then decomposed into phasic and tonic components through Continuous Decomposition Analysis (CDA)^92^. The phasic and tonic components represent the skin conductance response (SCR) and skin conductance level (SCL) portions of the signal respectively. Both artifact correction and CDA were performed in MATLAB (MathWorks, Natick, MA, USA) using the Ledalab-toolbox V3.4.9 (http://ledalab.de). The resulting SCL and SCR signals were imported into the R platform for statistical computing^88^ and analysed further together with behavioral and HRV data.

The impulse response function (IRF), which describes the temporal profile of each impulse of the phasic driver response and is used as the deconvolution kernel in the decomposition process, was estimated separately for each participant. The estimation was a two-step optimization process using gradient descent to minimize a compound error consisting of a weighted sum of negativity and indistinctness of the phasic driver. Indistinctness describes the sharpness of the impulses and negativity the number of negative values in the phasic driver.

#### EDA and HR1

One second aggregates of tonic and phasic signal components (SCL and SCR respectively) of the EDA, along with HR1, were used in similar manner to the HRV features above to address RQ2. In other words, RQ2 analysis procedure can be understood from the the section ‘ECG analysis’ above, substituting ${{\bf{x}}}_{i}^{SCL}$, ${{\bf{x}}}_{i}^{SCR}$, and ${{\bf{x}}}_{i}^{HR1}$ for the ECG features. In order to normalise the recording-specific participant-wise variation, SCR and SCL signals were z-scored within individual participant session data. This step is important to extract true relations due to shared experience, and avoid spurious correlations due to, e.g., unusually high basal skin conductance level. The resulting SPC values for SCR, SCL, and HR1, i.e., arithmetic means in correlations of the signals from collaborating pairs, are found in Table 1; p-values adjusted with Holm-Bonferroni correction.

### NetBeans with TMC plugin

Test-My-Code extension (TMC)^93^ for NetBeans integrated development environment (IDE) was used to track programming events. One-second granularity data collected by TMC was transformed into lists that contain programmer activities. The lists include task and user specific information on whether the source compiles, and information on whether the tests were executed and their outcomes or if the program was run.

Switching of roles was triggered by a pop-up window in TMC. Once the roles were switched (keyboard and mouse transferred), the new driver in the dyad clicked on a button in the pop-up to confirm that the switch was completed. Timestamps for each switch prompt and driver-confirm were used to track the role of each participant at all times.

Each computer in the classroom uses the same Network Time Protocol server, and the time is synchronized during the system startup. The computers were restarted at the start of each session to minimize clock drift.

#### Data integration

To match the data one-second granularity aggregates were computed. EDA data was resampled at 1 Hz and HRV parameters were repeated for each second due to one-second granularity of TMC output. A long-format data matrix was then constructed from the TMC, EDA, HR1 and background/self-report data, and analysed in R.

### Event based analysis

The TMC data was examined for distinguishable events relevant for the collaboration and pair dynamics. The types of events in which participants either tested the code or tried to run it were most likely to elicit EDA responses and were thus chosen for our analysis. The signal epoched was z-scored SCR signal. The extracted window were chosen to contain 10 seconds before and after each event. In total 1204 of such 20 second windows were collected. After filtering out the events that followed previous event in less than 10 seconds latency and keeping only the first of such events, 1006 events were included in subsequent analysis. The events were classified based on the participants role during the event and whether the event produced a positive or negative results, i.e., if the code could be compiled or the tests specific to the task were successful.

Due to the highly natural paradigm the number of run and test events depended fully on each pair. Thus the amount of these relevant events varied from zero to 28 in run events and zero to 22 in test events for a single collaborating pair. On average each pair had 27.1 run events and 8.9 test events. Regardless of varying data, a single average was computed for each participant in each category. This way, every participant contributed equally to the grand averages, except if the participant had zero instances in a particular category of events.

Specifically, when data was divided by role *or* by outcome, all participants did contribute an average. But when data was divided to the four role × outcome categories, for some categories the grand average did not include all participants because not everyone encountered, e.g., any failing events while driving. After weighting the contribution of each (possible) participant equally, the grand averages were computed for each category.

#### Differences in event based signals

Finally the correlation at 10 second lag for the SCR signals were examined. The method was applied to explore the temporal changes in individual signals. The autocorrelation was computed at 10 second delay to seek maximal delay correlations inside the analysis window. A grand average was computed similarly to above: autocorrelations were computed for individual averages for each participant in each condition and subsequently averaged; each individual autocorrelation contributed equally to the final average. This thus, searches signals for common autoregressive features.

#### Statistics for event based analysis

For statistical evaluation we first computed for each participant the difference between the averages in the roles (drive vs. navigate) and outcome (success vs. failure), respectively. We obtained 10000 samples by bootstrap resampling the subject-wise differences with replacement. We then randomly split these sampled time series to training and validation set of equal size. We computed the univariate naive 95% bootstrap confidence intervals using the training set. The univariate bootstrap confidence band have the interpretation for each time point that if the confidence interval at a given tome does not contain zero we can conclude with 95% confidence level that there is a significant difference^94^. However, these naive univariate intervals are not corrected for the effect of autocorrelation or multiple comparisons in the case of more than one time points. Therefore, the zero may occur outside the confidence intervals at some time point by random chance alone.

To correct for the effect of multiple confidence intervals we use the minimum width envelopes (MWE) by^67,74^. We used the greedy algorithm of Korpela and colleagues^67^ to find a MWE by using the training set and adjusted the parameter *k* by using the validation set by the procedure recommended by Korpela and colleagues^67^ such that 95% of the time series in validation set are fully within the MWE. As a result, we obtained MWEs such that a bootstrap resampled difference curve stays with 95% probability fully within the MWE. Thus, when comparing two conditions by constructing their difference curve, if the MWE at any point does not contain zero we can reject the null hypothesis that the roles or outcomes make no difference with a confidence level of 95%. This is analogous to univariate confidence intervals for individual points, i.e., a single instant in the time series. The MWE thus provides a 95% confidence band for difference signals, controlled for the family-wise error rate and corrected for the autocorrelation or other internal dependencies in the signal.

As an alternative, the same analysis of the difference curves can be done without the bootstrap by using permutation testing. Specifically, we obtain samples of difference curves by randomly permuting the roles (or outcomes), thus obtaining a set of difference curves that are exchangeable with the original data if the null hypothesis is true, i.e., the expected value for the difference curve is zero for all time points. We then compute a MWE for this set of randomly-sampled difference curves, analogously with the bootstrapped difference curves. The MWEs obtained by permutation testing are centered around zero difference, in contrast to the MWEs obtained by the bootstrap procedure which are centered around the observed difference. If the observed permuted difference curve is outside the MWE at any time point we can conclude that the roles or outcomes do not come from the same distribution.

The bootstrap approach is presented in the paper because it may be more intuitive, given that the MWEs are centered around the observed difference curves, similar to better-known naive confidence intervals. Whereas permutation testing has the advantage that theoretically it may be more robust, as it is closer to the more traditional hypothesis testing (such as presented, e.g., in Tables 1 and 2). For permutation testing the assumptions under which the bootstrap can be applied need not be considered; the situation is analogous to ‘traditional’ univariate confidence intervals which can be produced either with bootstrap (in which case they typically produce a reliability estimate for a parameter; if zero is not within the confidence interval we can conclude that the parameter in question is non-vanishing) or with permutation testing (in which case the confidence intervals are centred around zero and we can conclude that the parameter is non-vanishing if it appears outside the confidence interval). However, at least for the case discussed in our paper, the conclusions are unchanged independent of choosing the bootstrap or permutation based approach. The analysis with permutation testing is presented in the supplementary material.

### Data availability

Anonymized dataset that contains second granularity physiology and events used in the analysis of the current study is available in the institute web site at https://www.ttl.fi/avoindata/aineistot.
